# Ras GTPase-activating proteins control neuronal circuit development in barrel cortex layer 4

**DOI:** 10.3389/fnins.2022.901774

**Published:** 2022-09-16

**Authors:** Madhura S. Rao, Hiromi Mizuno, Takuji Iwasato, Hidenobu Mizuno

**Affiliations:** ^1^Laboratory of Multi-Dimensional Imaging, International Research Center for Medical Sciences (IRCMS), Kumamoto University, Kumamoto, Japan; ^2^Graduate School of Medical Sciences, Kumamoto University, Kumamoto, Japan; ^3^Laboratory of Mammalian Neural Circuits, National Institute of Genetics, Mishima, Japan; ^4^Department of Genetics, SOKENDAI The Graduate University for Advanced Studies, Mishima, Japan

**Keywords:** cerebral cortex, layer 4, sparse-cell labeling, RasGAP, neonates, thalamocortical axons

## Abstract

The cerebral cortex comprises a complex and exquisite network of neuronal circuits that is formed during development. To explore the molecular mechanisms involved in cortical circuit formation, the tactile somatosensory pathway that connects the whiskers and cortex of rodents is a useful model. Here, we analyzed the roles of Ras GTPase-activating proteins (RasGAPs) in the circuit formation in the somatosensory cortex layer 4 (L4). We suppressed the function of RasGAPs in L4 neurons using Supernova RNAi, a plasmid vector-based sparse cell gene knockdown (KD) system. The results showed disrupted dendritic pattern formation of L4 spiny stellate neurons on the barrel edge by RasGAP KD. Furthermore, the number of presynaptic boutons on L4 neurons was reduced by RasGAP KD. These results demonstrate the essential roles of RasGAPs in circuit formation in the cerebral cortex and imply that developmental changes in dendrites and synapses in RasGAP KD neurons may be related to cognitive disabilities in RasGAP-deficient individuals, such as patients with neurofibromatosis type 1.

## Introduction

Higher brain functions like cognition rely on a complex network of neuronal circuits. The tactile somatosensory pathway that connects the whiskers and cortex of rodents is useful for understanding the molecular mechanisms of neural circuit formation. The whiskers on the rodent’s snout receive tactile information such as the spatial orientation of its surroundings, target detection, and tactile discriminatory ability (Petersen, 2007; Fox, 2008). The rodent somatosensory barrel cortex layer 4 (L4) has a distinctive “barrel map” linked to the facial whisker pattern (Woolsey and Van der Loos, 1970). Thalamocortical (TC) axon terminals are clustered in the barrel center and excitatory neurons in L4 extend their dendrites toward the barrel center to receive inputs from TC axon terminals. These traits make the barrel cortex ideal for exploring the mechanisms of neocortical circuit development (Rao and Mizuno, 2021).

Previous studies have shown that Ras GTPase activating proteins (RasGAPs) play a role in neuronal circuit formation in the barrel cortex L4. For example, NF1*^flox/flox^*; hGFAP-Cre mice in which neurofibromin 1 (NF1), a RasGAP involved in neurofibromatosis type 1 disease, has been conditionally deleted in the cerebral cortex, fail to form the barrel patterning (Lush et al., 2008). Synaptic Ras GTPase activating protein (SynGAP) also plays a key role in the barrel pattern development (Barnett et al., 2006). However, the role of RasGAPs in the morphological development of individual L4 neurons remains unclear. Furthermore, because genes were knocked out in many cells of the brain in previous studies, the cell-autonomous functions of RasGAPs in the L4 circuit formation are obscure.

In this study, we analyzed the roles of RasGAPs in L4 circuit development. SynGAP and NF1 were suppressed in a sparse population of L4 neurons using *in utero* electroporation-mediated transfection of the Supernova RNAi system (Mizuno et al., 2014; Luo et al., 2016). In RasGAP knockdown (KD) neurons, dendritic patterning toward the barrel center was disrupted, and the presynaptic bouton number was reduced. These results suggest that RasGAPs are required for the development of L4 circuits in the developing somatosensory cortex.

## Materials and methods

### Experimental animals

ICR pregnant wild-type mice were used for E14 electroporation and post electroporation, the pregnant mice were kept on a heating pad at 37°C for 1–2 days and kept at room temperature in the animal cage. Further, the litter was maintained in the same cage after delivery. At the postnatal stage on P16, the mice pups of both genders were overdosed with pentobarbital and perfused with saline after observing the RFP-positive pups through a fluorescent binocular microscope (Leica). All experiments were conducted under the guidelines for animal experimentation of the Kumamoto University and approved by the animal experimentation committees.

### Supernova vector system

We used the Supernova-RNAi vector system for conditional gene suppression (Luo et al., 2016). This vector system consists of TRE-nCre (vector 1; 5 ng/μl) (Mizuno et al., 2014), CAG-loxP-stop-loxP-TurboRFP-ires-tTA-WPRE (vector 2; 1 μg/μl) (Mizuno et al., 2014) and CAG-loxP-stop-loxP-mir30 (0.5 μg/μl) (Matsuda and Cepko, 2007).

For knockdown experiments, template oligonucleotides containing shRNA sequences against the coding regions of SynGAP and NF1 and those scramble controls (Table 1) were ligated into the *Xho*I/*Eco*RI sites of pCAG-loxP-stop-loxP-mir30 vector (Matsuda and Cepko, 2007; Luo et al., 2016). Template oligonucleotides for knockdown vectors were designed according to the previous reports (Matsuda and Cepko, 2007; Parrinello et al., 2008) or using the RNAi designer (Block-iT, Invitrogen, CA, United States). For control vectors, oligonucleotides having low similarity with known mRNAs were designed using the scrambled sequence designer (GenScript, New Jersey, United States) and BLAST (NIH).

**TABLE 1:** Template sequences used to construct the RNAi expression vectors.

Target	Template sequence
SynGAP-1 (#17)	5′-cagaaggctcgagaaggtatattgctgttgacagtgagcgaCGTGCTCTGTATGAATCTGAGtagtgaagccacagatgtaCTCAGATTCATACAGAGCACGgtgcctactgcctcggaattcaaggggctactttag-3′
SynGAP-2 (#18)	5′-cagaaggctcgagaaggtatattgctgttgacagtgagcgaACATTGCTGACAGGCTGATCAtagtgaagccacagatgtaTGATCAGCCTGTCAGCAATGTgtgcctactgcctcggaattcaaggggctactttag-3′
NF1-1 (#19)	5′-cagaaggctcgagaaggtatattgctgttgacagtgagcgaCAAGCTAGAAGTGGCCTTGTAtagtgaagccacagatgtaTACAAGGCCACTTCTAGCTTGgtgcctactgcctcggaattcaaggggctactttag-3′
NF1-2 (#21)	5′-cagaaggctcgagaaggtatattgctgttgacagtgagcgaTTGCCTCAGTCAGCAATATGAtagtgaagccacagatgtaTCATATTGCTGACTGAGGCAAgtgcctactgcctcggaattcaaggggctactttag-3′
SynGAP-1 (scramble)	5′-cagaaggctcgagaaggtatattgctgttgacagtgagcgaAGGTGTCCGTTATTACCGAGTtagtgaagccacagatgtaACTCGGTAATAACGGACACCTgtgcctactgcctcggaattcaaggggctactttag-3′
SynGAP-2 (scramble)	5′-cagaaggctcgagaaggtatattgctgttgacagtgagcgaGATCGTCACGAATACGGTACTtagtgaagccacagatgtaAGTACCGTATTCGTGACGATCgtgcctactgcctcggaattcaaggggctactttag-3′
NF1-1 (scramble)	5′-cagaaggctcgagaaggtatattgctgttgacagtgagcgaACGGCATAAGTCGACGTATTGtagtgaagccacagatgtaCAATACGTCGACTTATGCCGTgtgcctactgcctcggaattcaaggggctactttag-3′
NF1-2 (scramble)	5′-cagaaggctcgagaaggtatattgctgttgacagtgagcgaGTCCGATAATCGAGACTTACTtagtgaagccacagatgtaAGTAAGTCTCGATTATCGGACgtgcctactgcctcggaattcaaggggctactttag-3′

Capital letters indicate the RNAi target sequences and their complementary sequences.

### *In utero* electroporation

*In utero* electroporation was conducted to transfect Supernova vector sets to L4 neurons as previously described (Mizuno et al., 2007, 2010, 2018, 2021). Pregnant mice were anesthetized at embryonic day (E) 14 with a combination of sodium pentobarbital (50 mg/kg body weight) and isoflurane gas (1.0–1.5% in air). A midline laparotomy was conducted to expose the uterus, and a pulled glass capillary was used to inject DNA into embryos. Square electric pulses (40 V, 50 ms, 1 Hz) were delivered to embryos three to five times using an electroporator (GEB14, Bex) and forceps-type electrodes (Bex). After electroporation, the abdominal wall and skin were sutured. Mice were allowed to recover on a heater (37°C).

### Histology and confocal microscopy

For histological analyses, mouse brains were fixed with 4% paraformaldehyde in phosphate buffer overnight and then transferred to 30% sucrose in PBS for 1 day. Tangential brain sections (100 μm thick) were made with a freezing microtome (Yamato Kohki, Saitama, Japan), then obtained sections were permeabilized and blocked in 0.2% Triton X-100/5% normal goat serum (Sigma, Missouri, United States) in PBS. Rabbit anti-VGluT2 (1:1,000, Synaptic Systems) and Alexa Fluor 488-conjugated goat anti-rabbit IgG (1:1,000, Invitrogen, CA, United States) antibodies were used to visualize the barrel arrangement in obtained sections. Fluorescent images were obtained using a SP8LS confocal microscope (Leica). The obtained images from confocal microscopy observation were assessed using LasX software (Leica), Image J software with Fiji (Schindelin et al., 2012), Imaris software (Bitplane, Belfast, United Kingdom), and Microsoft Excel.

Dendritic pattern analyses (Figure 2) were performed as previously described (Mizuno et al., 2014). For the orientation bias index (OBI) analysis (Figure 2), we selected only sparsely labeled spiny stellate neurons with clear dendritic morphologies on the barrel edge. The barrel edge was defined as the edge of TC axon clusters labeled with vGluT2 immunostaining. Neurons with cell bodies located at the edge of sections were excluded from analyses. For spine and bouton analyses, secondary dendritic branches located inside the barrels were used considering barrel edge neurons and center neurons, and only one branch from one neuron was used (the branch number is equal to the neurons number). Spine and bouton numbers were counted using 3D models created from sequential optical sections by Imaris software. All spine types (stubby, thin, and mushroom) with more than 1 μm in length were defined as spines. For bouton analysis, vGluT2 signals with more than 1 μm in diameter and attached on spines were defined as boutons.

### Statistical analysis

All results are presented as mean ± standard error. The significance of the differences was analyzed using a Mann–Whitney *U* test with Bonferroni correction for the dendrite analysis (Figure 2) and a two-tailed Student’s *t*-test with Bonferroni correction for the spine and puncta analysis (Figure 3). All *p*-values < 0.05 were considered statistically significant.

## Results

### Ras GTPase-activating proteins are required for dendritic development of layer 4 neurons

**FIGURE 1:**
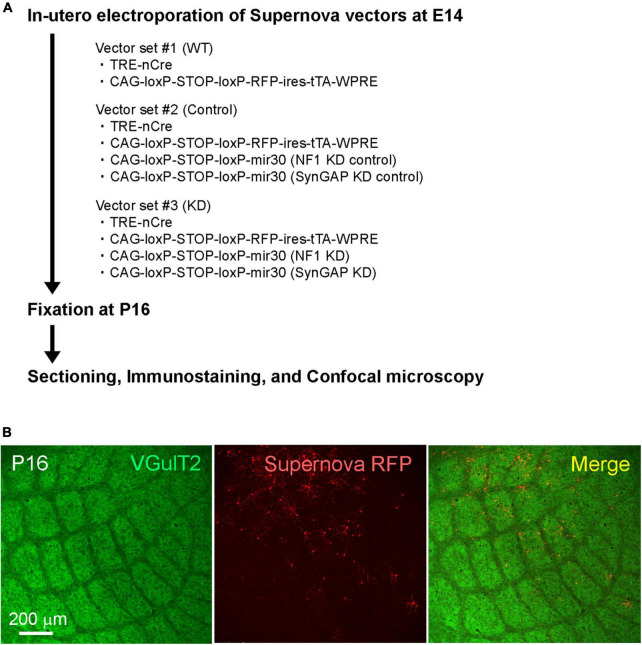
**(A)** Schematic of the Ras GTPase-activating protein (RasGAP) knockdown experiments. **(B)** Example confocal images of VGluT2-immunostained tangential sections obtained from Supernova-electroporated P16 mice.

To study the function of RasGAPs in barrel circuit formation, we transfected the Supernova vector system for conditional gene KD (Supernova RNAi) (Luo et al., 2016) to L4 neurons using *in utero* electroporation. In this study, we focused on NF1 and SynGAP for RasGAP KD, because previous studies reported these two molecules were involved in the barrel pattern formation (Barnett et al., 2006; Lush et al., 2008). Supernova RNAi consists of TRE-Cre (vector 1; TRE: tetracycline response element; Cre: site-specific recombinase), CAG-loxP-stop-loxP-TurboRFP-ires-tTA-WPRE (vector 2; CAG: CAG promoter; loxP: Cre recombinase target sequence; stop: transcriptional stop sequence; TurboRFP: red fluorescent protein variant; ires: internal ribosome entry site; tTA: tetracycline transactivator; and WPRE: woodchuck hepatitis virus posttranscriptional regulatory element), and Cre-dependent KD vectors (Figure 1A and Table 1). TurboRFP expression in a sparse population of transfected neurons was enabled by the positive feedback of the tTA-Cre cycle between vectors 1 and 2 (Mizuno et al., 2014; Figure 1B). In this system, KD RNAs were expressed in RFP expressing cells (Luo et al., 2016).

**FIGURE 2:**
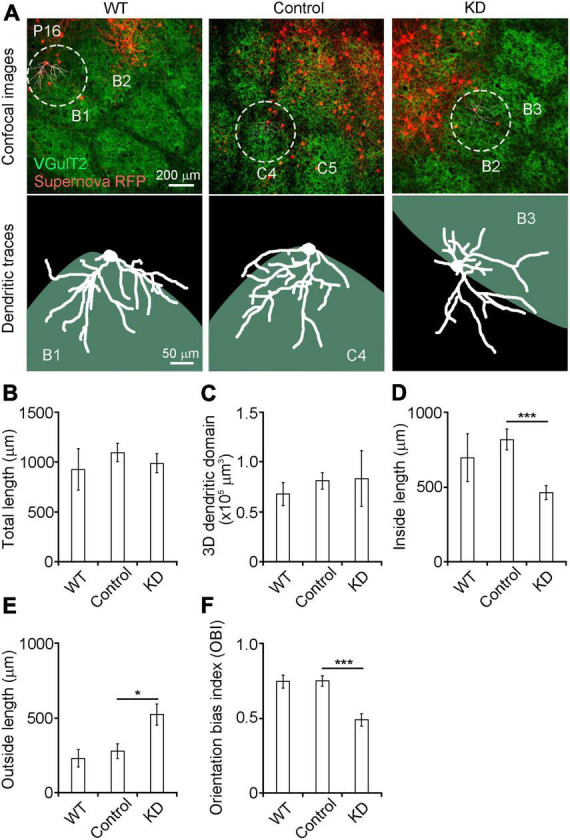
**(A)** Dendritic orientation was impaired in RasGAP-suppressed neurons Top images: confocal images obtained from P16 mice (left: wild-type, middle: RNAi control; right RasGAP knockdown). Bottom images: dendritic tracings of Supernova RFP-labeled L4 neurons. **(B–F)** Quantitative analysis of dendritic morphology (WT, 7 cells from 5 mice; control, 12 cells from 8 mice; KD, 13 cells from 6 mice). The orientation bias index was calculated by dividing the inside dendritic length by the total dendritic length. *p < 0.05, ***p < 0.001.

**FIGURE 3:**
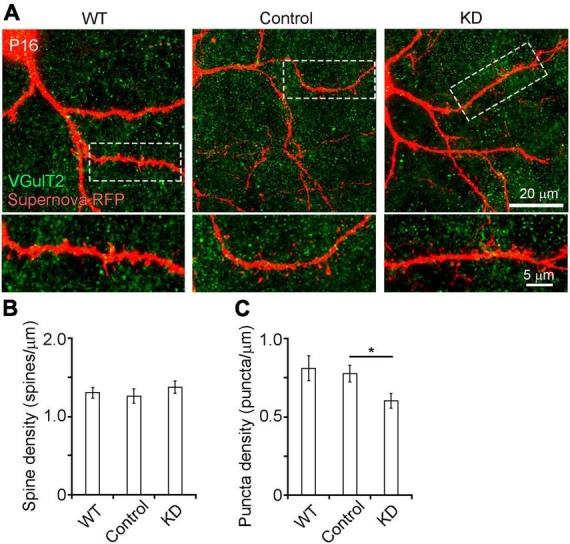
**(A)** Spine and presynaptic bouton analysis of RasGAP-suppressed neurons at P16 by high magnification confocal imaging (63× oil imaging). **(B,C)** Quantitative analysis of spine **(B)** and presynaptic bouton **(C)** density in secondary branches of L4 neuron dendrites located inside the barrels (WT, 28 branches from 8 mice; control, 22 branches from 13 mice; KD, 31 branches from 16 mice). *p < 0.05.

We analyzed the roles of RasGAPs in the dendritic pattern formation of spiny stellate neurons on the barrel edge at P16 when the dendritic branch pattern of these neurons was essentially mature (Figure 2A). For KD of RasGAP, we mixed four KD vectors with vectors 1–2 (Table 1). We first compared wild-type (WT) and control (RasGAP RNAi scrambled sequences) neurons, as the expression of the four types of control RNAi could affect the normal development of L4 neurons, although the control sequences have low similarity with the known mRNAs. We found that the total dendritic length, the 3D dendritic domain, and dendritic lengths inside and outside the barrel did not differ between WT and control neurons (Figures 2B–E). Furthermore, the OBI, defined as the ratio of the inside length to the total length, was similar between WT and control neurons (Figure 2F). These results support that the control vectors do not affect the dendritic pattern formation of L4 spiny stellate neurons.

Next, we compared dendritic morphology between control and RasGAP KD spiny stellate neurons. The total dendritic length and the 3D dendritic domain did not differ between control and RasGAP KD neurons (Figures 2B,C). However, the dendritic length inside the barrel was shorter, and, the length outside the barrel was longer in KD cells compared with control cells (Figures 2D,E; inside: *p* = 0.003; outside: *p* = 0.030). Consequently, the OBI in KD cells was smaller than that in control cells (Figure 2F, *p* < 0.001). These results suggest that RasGAPs are required for the dendritic pattern formation of L4 spiny stellate neurons on the barrel edge.

### Ras GTPase-activating proteins are required for presynaptic bouton formation

Ras GTPase-activating proteins may also be involved in synapse formation in L4 neurons. We first analyzed the effect of RasGAP KD on spine formation (Figure 3A). High magnification confocal imaging revealed that spine density on the secondary dendritic branches of control cells was not different from that of WT and KD cells (Figure 3B). To examine the role of RasGAPs in presynaptic structure formation, we counted the number of presynaptic boutons which are mainly originated from the thalamocortical axon terminals, which were labeled with anti-VGluT2 immunostaining, on the secondary dendritic branches of L4 neurons. Although the number of presynaptic boutons did not differ between control and WT cells, KD cells had a smaller bouton number than control cells (Figure 3C, *p* = 0.029). These results suggest that RasGAPs are required for presynapse formation.

## Discussion

In this study, we analyzed the functions of RasGAPs in the developing barrel cortex L4 using the Supernova RNAi system. We found that dendritic patterning toward the barrel center was disrupted in RasGAP KD L4 neurons. Previous studies have reported the genes involved in circuit formation in L4 (Wu et al., 2011; Rao and Mizuno, 2021), and dendritic pattern disruption in RasGAP KD neurons is similar to the phenotype in L4 cells of several gene knockout animals. Among them, the N-methyl-D-aspartate (NMDA) receptor is a candidate molecule involved in RasGAP signaling. The cell-autonomous function of the NMDA receptor is related to the dendritic pattern formation of L4 neurons (Espinosa et al., 2009; Mizuno et al., 2014). Furthermore, prior studies have shown that the barrel pattern is disrupted in cortex-specific NMDA receptor-deficient, SynGAP knockout, and cortex-specific NF1 knockout animals (Iwasato et al., 2000; Barnett et al., 2006; Lush et al., 2008). In the hippocampus, RasGAP activity is regulated by the NMDA receptor (Komiyama et al., 2002), and NF1 and NMDA receptor signaling is involved in spine structural plasticity (Oliveira and Yasuda, 2014). These results indicate that NMDA receptor-RasGAP signaling may also be involved in the dendritic pattern formation of L4 neurons in the barrel cortex.

The present study showed that the spine number of L4 neurons was not affected by the suppression of RasGAPs. However, the number of vglut2-labeled presynaptic buttons, which could be more reliable if confirmed with co-localization of VGulT2-Synapsin, has decreased. These results suggest that RasGAP in L4 cells retrogradely regulates presynapse formation in TC axons. This signaling could be mediated by retrograde molecules (Regehr et al., 2009). For example, semaphorin derived from Purkinje cells regulates the formation of climbing fiber synapses in the developing cerebellum (Uesaka et al., 2014). Molecular interactions between RasGAPs and candidate signaling molecules like the NMDA receptor could be assessed through experiments using co-immunoprecipitation, rescue experiments, and gene-trapping assays.

The findings of this study also provide novel insights for the pathophysiology of brain disease due to dysfunction of RasGAPs. NF1, one of the RasGAPs encoding neurofibromin, is the causative gene of neurofibromatosis type I. Previous studies have reported learning disabilities in 30–65% of children with neurofibromatosis type I (North, 2000) and memory impairment in neurofibromatosis type I model mice (Costa et al., 2002). However, little is known about neuronal circuit level changes in NF1 deficiency. Our findings suggest that changes in dendritic and synaptic development in the cerebral cortex are related to the cognitive disabilities commonly seen in patients with neurofibromatosis type I.

## Data availability statement

The raw data supporting the conclusions of this article will be made available by the authors, without undue reservation.

## Ethics statement

This animal study was reviewed and approved by the Animal Care and Use Committee, Kumamoto University.

## Author contributions

HidM conceived the study. MR and HirM performed histological experiments. HirM, TI, and HidM contributed to the plasmid construction. MR and HidM analyzed the data and wrote the draft. All authors read the manuscript.
